# PDE4D regulates Spine Plasticity and Memory in the Retrosplenial Cortex

**DOI:** 10.1038/s41598-018-22193-0

**Published:** 2018-03-01

**Authors:** Karsten Baumgärtel, Andrea Green, Diana Hornberger, Jennifer Lapira, Christopher Rex, Damian G. Wheeler, Marco Peters

**Affiliations:** 1 Dart Neuroscience, LLC, 12278, Scripps Summit Drive, San Diego, CA 92131 USA; 2 Afraxis Inc., 6605, Nancy Ridge Rd. Suite 224, San Diego, CA 92121 USA

## Abstract

The retrosplenial cortex (RSC) plays a critical role in episodic memory, but the molecular mechanisms governing plasticity in this structure are poorly understood. Diverse studies have demonstrated a role for RSC in acquisition, early consolidation and retrieval similar to the hippocampus (HC), as well as in systems consolidation similar to the anterior cingulate cortex. Here, we asked whether established molecular and structural substrates of memory consolidation in the HC also engage in RSC shortly after learning. We show striking parallels in training induced gene-activation in HC and RSC following contextual conditioning, which is blocked by systemic administration of an NMDA receptor antagonist. Long-term memory is enhanced by retrosplenial and hippocampal knockdown (KD) of the cAMP specific phosphodiesterase *Pde4d*. However, while training *per se* induces lasting spine changes in HC, this does not occur in RSC. Instead, increases in the number of mature dendritic spines are found in the RSC only if cAMP signaling is augmented by Pde4d KD, and spine changes are at least partially independent of training. This research highlights parallels and differences in spine plasticity mechanisms between HC and RSC, and provides evidence for a functional dissociation of the two.

## Introduction

Since the first description of amnesia in a patient with retrosplenial lesion by Valenstein and colleagues almost 30 years ago^1^, much of the insight on the role of the retrosplenial cortex (RSC) in memory comes from studies examining its activation, or the effect of its inactivation in animal models^2^. These studies have revealed that RSC is activated by contextual fear conditioning (cFC)^3^; and training in spatial learning tasks such as the Morris Water Maze^4,5^, and that its inactivation or lesion impairs performance^6–11^, These and other studies demonstrate the necessity of the RSC for contextual and spatial tasks in rodents, findings consistent with clinical observations^1,2^. Recent evidence suggests that this necessity derives from a direct role in learning and memory, such as linking sensory stimuli during learning^12^, consolidation^5,13–16^, or retrieval of memory^10,17^. Ectopic expression of cAMP-response binding protein (CREB) in RSC augments spatial memory^5^, while inhibition of protein synthesis and dopamine D1 receptors impairs^13^ - suggesting that well known molecular mechanisms of memory consolidation in the hippocampus (HC) may be conserved in RSC. Here, we aimed to systematically interrogate whether NMDA-receptor dependent gene-activation, reliance on cAMP-signaling, and structural plasticity as established processes of memory consolidation in the HC occur in parallel in the RSC. We demonstrate that the training induced transcriptional response in HC and RSC are indistinguishable and are N-methyl-D-aspartate receptor (NMDA-R) dependent. We then focus on cyclic adenosine monophosphate (cAMP) signaling, which is fundamental to cognitive processes and memory in HC but remains unexplored in the RSC. cAMP levels are tightly regulated by phosphodiesterases (PDEs) and the function of Pde4 in HC has been extensively studied. We replicate the finding that *Pde4D* knockdown in HC enhances memory^18^, and show that training induces changes in mature spines in HC that are augmented by *Pde4D* KD. This is contrasted by findings in the RSC. While *Pde4D* knockdown in RSC also enhances memory, there are no training- but only *Pde4D* KD-dependent spine changes.

## Materials and Methods

### Mice

All animal work adhered to procedural protocols approved by the IACUC committee of Dart Neuroscience, LLC. Methods were performed in accordance with the relevant guidelines and regulations. We used male C57Bl/6 mice (Taconic Farms) for RNAseq, immunohistochemistry, rolipram, and MK801 studies; and male C57Bl/6 × 129SvTac hybrid mice (Taconic Farms) for *Pde4d* knockdown and spine imaging studies. Mice were housed in cages of four, maintained on a 12 hr light/dark schedule (PST: 6 am to 6 pm; 7 am to 7 pm during day light savings), and allowed *ad libitum* access to food and water. Experiments were conducted on 3–6 month old male mice during the light phase. Experiments and analysis were performed blind to the treatment condition of each mouse.

### Contextual fear conditioning task (cFC)

Mice were trained and tested in conditioning chambers fitted with a stainless steel grid floor through which footshocks can be delivered (Coulbourn Instruments, Pennsylvania, USA; and Med Associates Inc, Vermont, USA). Training consisted of placing the mice in the chamber and after 120 sec delivering either two (to induce a weak memory) or five (strong memory) electrical footshocks (2 sec duration; 0.6 mA for C57Bl/6 mice, 0.2 mA for hybrid mice) separated by a 60 sec inter-trial interval (ITI). Mice were returned to the home cage 30 sec after the final footshock. Different mouse lines show differential sensitivity to the shock in the cFC task and shock intensities were selected to generate comparable freezing. Memory was assessed at the stated time after training by re-placing the mice into the training context and calculating the percentage of time spent freezing during 3 mins. Freezing behavior was defined as the complete lack of movement except for respiration for at least 1 sec, and was measured automatically using motion detection software (Freezeframe by Coulbourn Instruments, Pennsylvania, USA; and Video freeze by Med Associates Inc, Vermont, USA). For the induction of the training-induced transcriptional response, an additional group was included that was placed into the context for the same duration but did not receive footshock.

### Object exploration training (OE)

Mice were habituated for 5 mins in square open field chambers (40 cm W × 40 cm D × 35 cm H) filled with cobb bedding under dim light on two consecutive days. Training involved placing two identical objects along one midline of the chambers. Mice were trained for 15 mins and allowed to explore the objects freely.

### Tissue collection

For mRNA preparation, mice were anesthesized with isofluorane until all reflexes were lost and they were then sacrificed by decapitation. The brain was extracted and immediately dropped in ice cold PBS. After 2–5 mins in PBS a section of the area between approximately bregma −0.5 mm and −3.5 mm was taken using a coronal brain matrix, and the RSC and HC were dissected from this section and directly frozen on dry ice. Tissue was kept frozen at −80 °C until RNA extraction. Tissue was sampled from trained and naïve mice in parallel for each time-point and condition tested.

### Quantitative RT-PCR

RNA was isolated using the QIAgen RNeasy kit (Qiagen) according to the manufacturer’s specifications. cDNA was generated using Taqman Fast Advanced Master Mix (ThermoFisher Scientific), and quantitative PCR (qPCR) conducted with Taqman probes for the immediate early genes, *Arc* (Mm00479619_g1), *Fos* (Mm00487425_m1)*, Npas4* (Mm00463644_m1), *Pde4d* (Mm00456879_m1) as well as the reference housekeeping gene *Gapdh* (Mm03302249_g1) using a StepOnePlus Real-Time PCR System (ThermoFisher Scientific). qPCR reactions were run in triplicate and the CT values were averaged. Data was normalized to *Gapdh* and ΔΔCT values were determined relative to a group of untrained mice taken directly from the home-cage and sampled at time-point 0.

### RNA-Seq

RNA was sampled as above and concentration and quality were determined using a NanoDrop 8000 (ThermoFisher Scientific) and Bioanalyzer (Agilent), respectively. RNA-Seq libraries prepared with non-stranded NEBNext® Ultra™ RNA Library Prep Kit for Illumina® (NEB) using random hexamers. Illumina-compatible adaptors and unique indexes were added and DNA fragments were amplified with 12 rounds of PCR. Ampure beads (Beckman Coulter) were used for size exclusion. 3–20 pM of each library underwent paired end (2 × 50) sequencing on a HighSeq 2000 (Illumina) with 8 libraries multiplexed per lane. FastQ files were aligned to the Enseml transcriptome via Bowtie and differential expression was determined using DE-Seq2. To avoid complications from false positives, we set a p-value cut-off of p < 0.01 for significance and conducted two completely independent experiments (n = 4 mice per condition/experiment). Only those genes that were called in both experiments (concordant) were considered confirmed.

### Immunohistochemistry

Seventy-five minutes after cFC training, mice were transcardially perfused with PBS for 2 mins followed by 4% PFA for 5 mins, and brains post-fixed overnight in 4% PFA. Free-floating 50 μm coronal sections were prepared using a vibratome. Sections were blocked and permeabilized with 0.1% BSA, 0.2% Triton X, 10% normal goat serum overnight and stained with anti-FOS-1 rabbit polyclonal antibody (Cell signaling; 1:750). Sections were then stained with Alexafluor 568 goat anti-rabbit secondary antibody (Molecular probes; 1:500) using the same blocking solution, and - after a series of washes with PBS - with DAPI (Invitrogen; 1:1000). Slide mounted sections were imaged using a Zeiss LSM 780 confocal microscope using a 10× objective and Zen software. Quantification was performed using the Metamorph software (Molecular Devices, Sunnyvale, CA) by autocounting of number of FOS positive cells per area and creating averages of at least 4 sections per mouse.

### Vehicles and surgical procedures for *in vivo* RNA interference

shRNA targeting *Pde4D* was expressed from a U6 promoter and a GFP reporter gene (to monitor *in vivo* transduction by AAV5) was expressed from a CBA (chick beta-actin) promoter contained in the same AAV5 virus. shRNA targeting GFP was used to control for non-specific effects of viral transduction and shRNA expression on memory formation. Efficiency to knock-down *Pde4d* was confirmed in cultured mouse hippocampal neurons. For virus injection, mice were anesthetized with a Ketamine/Xylazine (10:1, at a dose of 100 mg/kg Ketamine) anesthetic, core body temperature maintained throughout the surgery using a heat blanket, and an ophthalmic ointment applied. Bregma and lambda were leveled to the same plane, as were two points 2 mm of each side of the midline. For HC injections, holes were drilled at stereotactic coordinates AP = −1.5 mm, Lateral = ±1.5 mm and the injection cannula was lowered 1.75 mm below the surface of the skull. One µl of virus was injected bilaterally at a rate of 0.5 µl/min, and after 1 min the cannula was pulled up to −1.5 mm and another 1 µl was injected. After an additional 1 min the cannula was removed. For RSC, holes were drilled at stereotactic coordinates AP = −4.5 mm, Lateral = ±0.2 mm. The cannula was inserted at a 22 degree angle and 0.5 µl virus injected at 3.6, 2.6 and 1.6 mm ventral to bregma (bilaterally) at a rate of 0.5 µl/min. After an additional 1 min the cannula was removed. After injection, a thin layer of bone wax was applied to limit efflux from the injection site and drying of the tissue. The skin was closed above the scalp, post-surgery care was provided, and mice were allowed to recover for 10–14 d prior to experimentation. For intrahippocampal rolipram injections, mice were implanted with a 33-gauge guide cannula bilaterally into the dorsal hippocampus (coordinates: A = −1.8 mm, L = +/−1.5 mm to a depth of 1.2 mm^19^. Five to seven days after recovery from surgery, mice were trained in the cFC task and rolipram or vehicle alone (1μL per hemisphere) was infused into the hippocampus 5 mins afterwards. The vehicle was comprised of 1% DMSO and 99% PBS (pH7.2).

### Spine analysis

24 hrs after cFC training, mice were transcardially perfused with first PBS for 30 sec and then 4% PFA for 1.5 mins, followed by 10 min postfixation. Brains were stored in PBS and transferred to Afraxis (San Diego, USA) for analysis. Brain tissue was sectioned using a tissue vibratome (Leica VT1000) to collect 300 μm sections from the anterior to posterior extremes of each brain. Ballistic dye labeling (DiI and DiO) was performed using protocols described previously^20^, after which sections were slide mounted and coverslipped. Laser‐scanning confocal microscopy was conducted (Olympus FV1000; 63x, 1.42 NA) to scan individually labeled neurons at high resolution (0.10 × 0.10 × 0.33 μm voxels). Target neurons were identified by anatomical location and cell morphology. Microscopy was performed blind to experimental conditions. Blind deconvolution (AutoQuant) was applied to raw three‐dimensional digital images which were then analyzed for spine density and morphology. Individual spines were measured manually for head diameter, length, and neck thickness from image Z-stacks using custom-built Afraxis ESP software. 5–7 cells (RSC) or 7–8 cells (HC) were sampled per mouse, and each dendrite was analyzed by 3 independent analysts. Afraxis automated image assignment and spine measurement and classification criteria are described previously^21^ and follow an established paradigm^22^. For spine density and spine morphological classification, data across analysts were averaged to report data for each dendrite. All Afraxis experimenters were fully blinded to treatment conditions during the collection, assembly, and interpretation of the data.

### Statistical analysis

Behavioral, RT-PCR, and spine imaging data were analyzed by unpaired t-test (where appropriate) or by ANOVA, followed by planned comparisons to interrogate differences amongst individual treatment groups. RNAseq data were analyzed by linear regression to determine correlated responses. Error bars represent s.e.m. in all figures.

## Results

### Learning engages NMDA receptor-dependent and highly correlated transcriptional responses in RSC and HC

**Figure 1:**
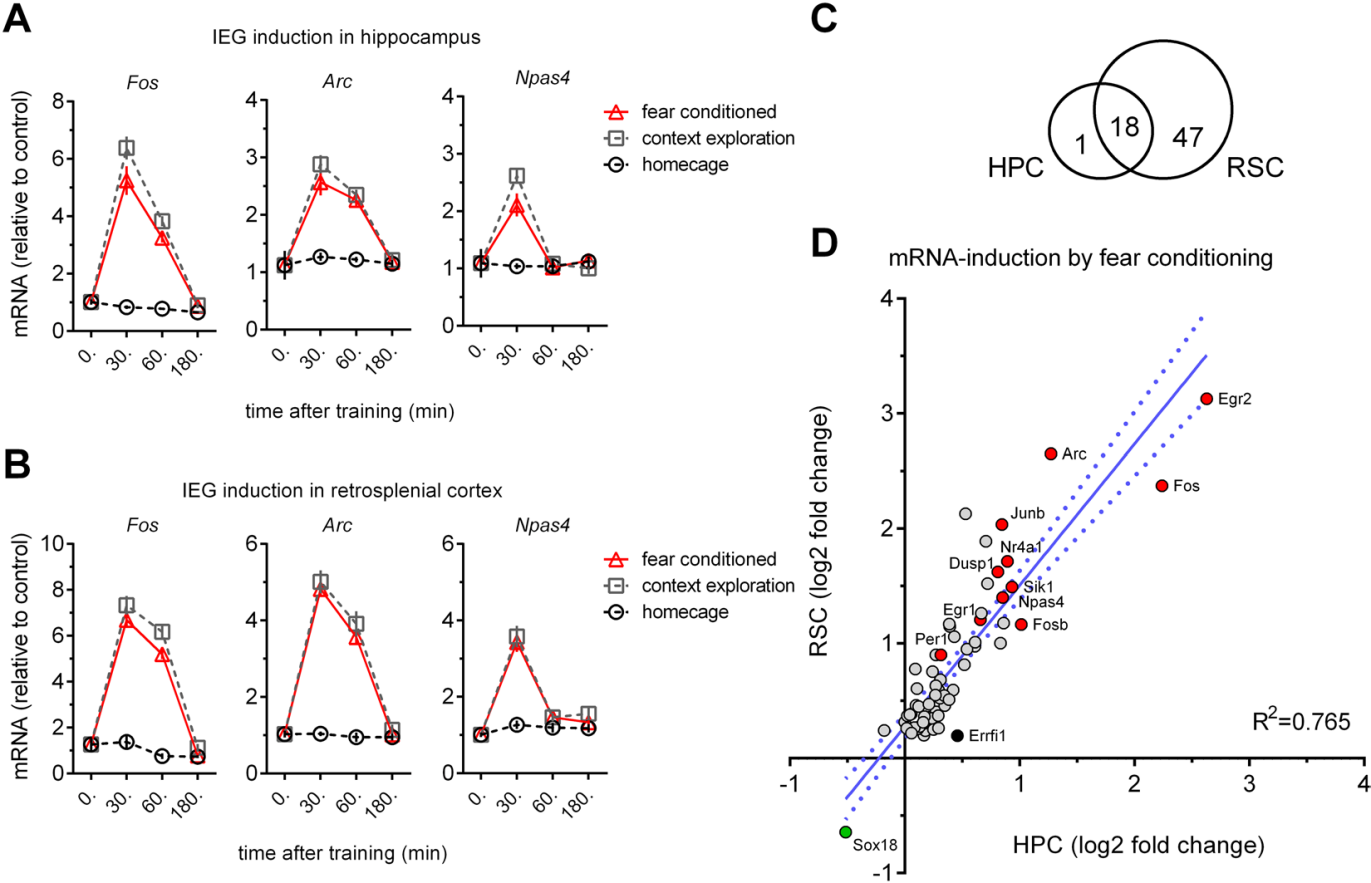
Gene-responses following cFC are highly correlated in hippocampus (HC) and retrosplenial cortex (RSC). (**A**) In HC, IEG mRNA levels (*Fos*, *Arc* and *Npas4*) as detected by qPCR are strongly increased 30 mins (all three genes) and 1 hr (*Fos*, *Arc*) after cFC training, and return to baseline within 3 hrs. (**B**) The time-course of IEG induction observed in HC is recapitulated in RSC (n = 8 mice per group for HC and RSC). (**C**) Venn diagram showing the number of confirmed hits of two replicate RNAseq studies comparing mRNA expression in naïve and fear conditioned mice. All but one gene (*Errf1*) found in HC are detected in RSC. (**D**) Correlation of mRNA induction by fear conditioning in HP and RSC. The gene-responses of the 66 confirmed hits in RSC and HC are highly correlated. The greater number of calls in RSC is explained by an overall larger gene-response in this structure.

**Figure 2:**
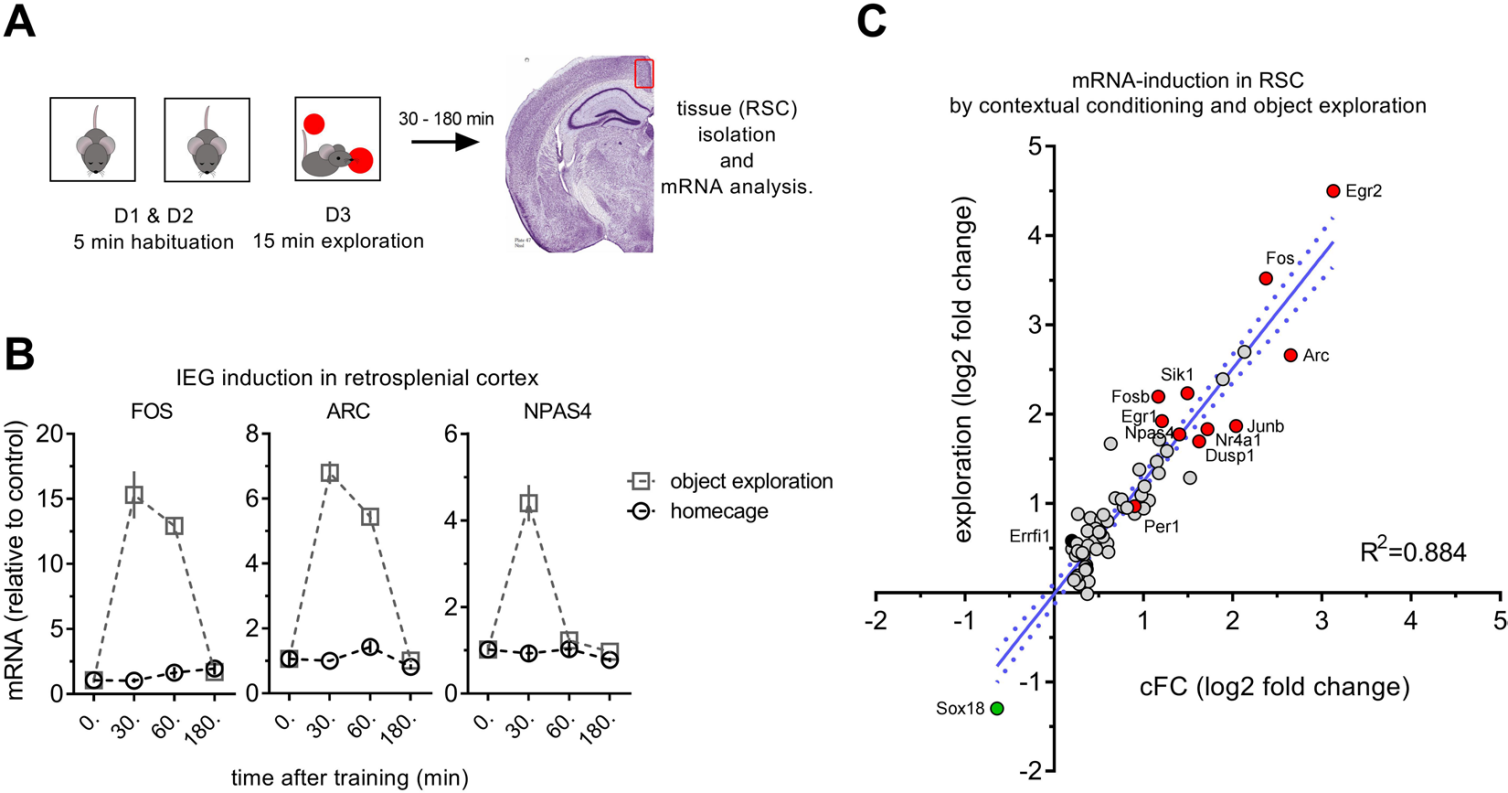
Object exploration (OE) training strongly activates RSC. (**A**) The training protocol used to test the effect of exploration on gene expression involves 2 d of habituation to the training boxes and a 15 min exposure to the objects on training day, a procedure that will produce a memory for both the objects (object recognition memory) and their spatial location (object location memory). (**B**) In RSC, IEG mRNA levels (*Fos*, *Arc* and *Npas4*) as detected by qPCR are strongly increased 30 mins (all three genes) and 1 hr (*Fos, Arc*) after OE training, but return to baseline within 3 hrs thereby recapitulating the time course seen after cFC training (n = 8). (**C**) Correlation of RSC induction of the 66 genes shown in Fig. 1d after cFC and OE training. The OE induced gene expression changes in RSC closely recapitulate those seen 30 mins after cFC training.

Learning induced transcriptional responses and *de novo* synthesis of proteins are critical for memory consolidation^23^. We therefore characterized the time-course of immediate early gene (IEG) expression in RSC after learning, and asked how the response relates to the hippocampus. We trained mice with five trials in the cFC task to induce a strong memory, and compared gene-expression at 30, 60, and 180 min after training to naïve (home-cage) controls (Fig. 1a/b). As expected, a marked upregulation of the IEGs *Fos*, *Arc*, and *Npas4* occurs within 30 mins of training in the hippocampus, which lasts for up to 60 mins after training for *Fos* and *Arc*, and then returns to baseline within 3 hours (Fig. 1a). This profile of IEG induction is recapitulated by context exposure in the absence of shock, in accordance with previous findings (for example, see ref.^17^). This dynamic is mirrored in RSC of the same animals (Fig. 1b). This suggests that the formation of a contextual representation or the detection of novelty (rather than the association of context and shock) activates IEG expression in RSC and HC. This conclusion is supported by IEG induction after exposure to two objects in a familiar open field environment, a setup commonly used for testing object recognition and object location memory (OE, Fig. 2a). This alternative (non-aversive) learning paradigm similarly increases IEG levels in RSC, but a larger *Fos* response was elicited than in the cFC task (Fig. 2b). Thus, IEGs are induced in both HC and RSC within minutes of learning, returning to baseline after one (*Npas4*) to three (*Fos*, *Arc*) hours. To further characterize the induction of IEGs after training at the cellular level, we quantified the number of FOS protein-positive cells in RSC and the hippocampal subfields 75 mins after cFC training. Both cFC training and context exposure in the absence of foot-shock result in a doubling of FOS positive cells in RSC and hippocampal CA1 and CA3, but not in dentate gyrus (**RSC**: ANOVA F(11, 2) = 40.27, p < 0.0001; naïve 28.70 +/− 2.77 vs context 61.57 +/− 2.27 vs trained 48.14 +/− 2.74; **CA1**: ANOVA F(11, 2) = 30.87, p < 0.0001; 7.57 +/− 0.34 vs 13.68 +/− 0.99 vs 15.01 +/− 0.66; **CA3**: 3.84 +/− 0.13 vs 8.28 +/− 0.56 vs 8.76 +/− 0.35; **DG**: *not significant*, 6.19 +/− 0.62 vs 7.15 +/− 0.48 vs 6.84 +/− 0.54).

*Fos*, *Arc* and *Npas4* are common IEGs that show robust induction, but they may not be representative of the transcriptional response as a whole, which relies on multiple transcription factors and second messenger cascades. To generate a more complete picture of the relationship between gene-induction in RSC and HC we processed samples taken 30 mins after cFC training for RNA-Seq We used a p-value cutoff of p < 0.01 in individual experiments and only considered genes as true positive hits if they were significant in two independent studies. Of the 19 genes up-regulated by training in HC, 18 are also found to be significant with these strict criteria in RSC (Fig. 1c). In addition to the above described genes *Fos*, *Arc*, and *Npas4*, we identify (amongst other genes) *Fosb, Junb*, *Dusp1*, *Sik1*, *Nr4a1* (Nur77), *Ier2*, *Btg2*, *Egr1* (Zif268), *Egr2*, and *Egr4* – induction of which was previously described in HC but has not been characterized in RSC. Only one gene (*Sox18*) is downregulated, and 47 additional genes are significantly upregulated by training in RSC. Our data do not indicate that there are genes specific to RSC, but rather that the magnitude of the mRNA level increase in RSC is larger allowing more genes to meet statistical significance. Indeed, linear regression analysis of genes significantly regulated in RSC shows that gene expression changes in the HC are strongly correlated even if individual genes do not meet the significance criteria (β: 1.229, α: 0.278, R^2^: 0.765, p < 0.0001; Fig. 1d). *Errfi1* is significantly increased in HC but not RSC of fear conditioned mice. Again, this does not suggest that this is a HC-specific response, but just that the increase in *Errfi1* was not statistically significant. In fact, this gene is significantly increased in RSC after object exploration. More generally, object exploration produces gene expression changes almost identical to those found in the fear conditioning (FC) studies (β: 1.261, α: −0.014, R^2^: 0.884, p < 0.0001; Fig. 2c). Thus, the transcriptional response at 30 min after learning is highly correlated in HC and RSC although more genes are detected in RSC due to better ‘signal to noise’, and it is similar in mice trained in the cFC and object exploration tasks.

**Figure 3:**
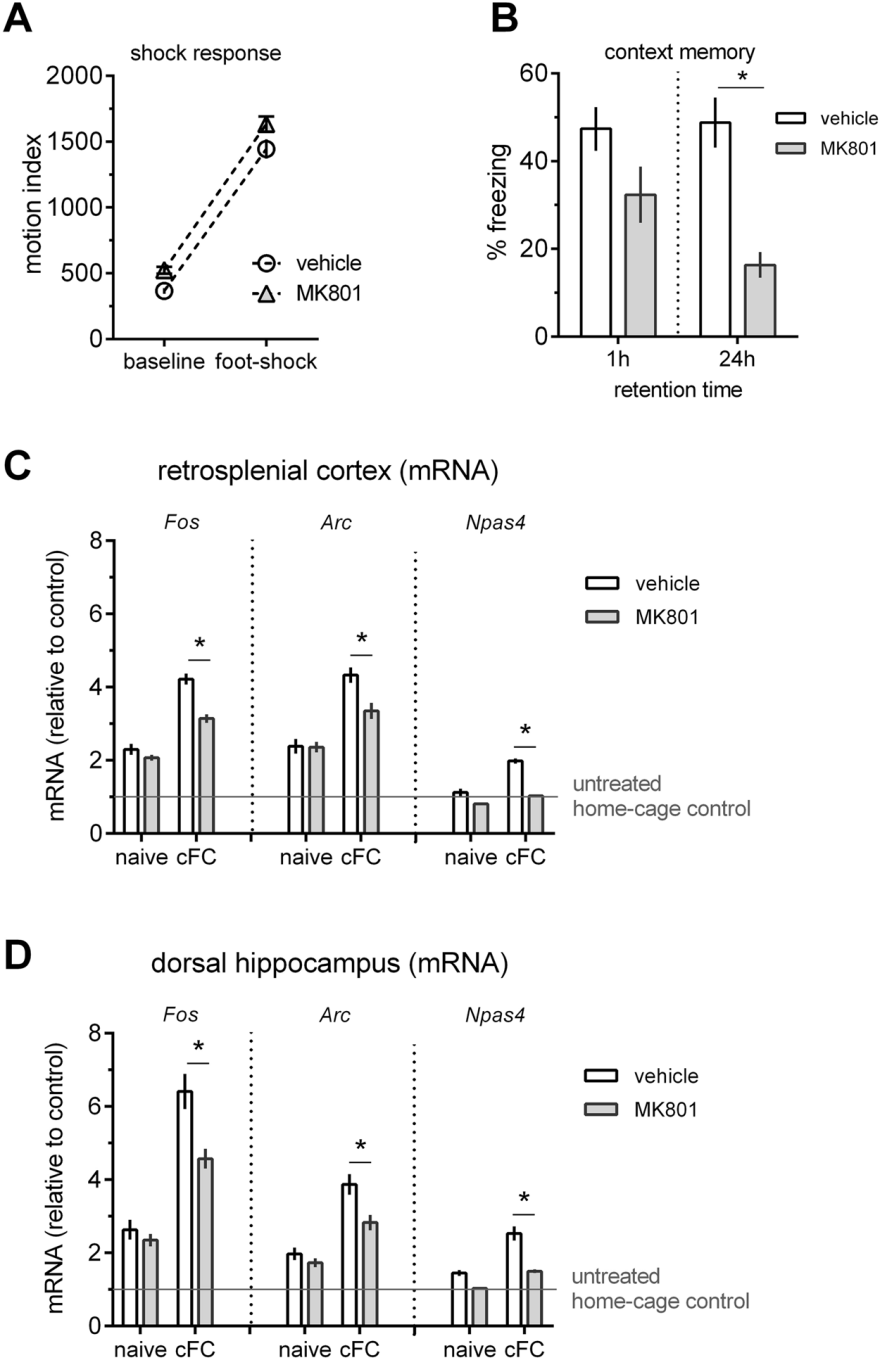
Blocking NMDA-R impairs memory and blocks IEG induction in HC and RSC. MK801 (0.1 mg/kg, i.p.) injected 30 mins before cFC training does not affect the response to foot-shock during conditioning (**A**), but it significantly impairs memory when tested 24 hrs but not 1 hr after training (**B**), n = 8 per treatment group and time-point). The same dose blocks training-induced IEG induction (*Fos*, *Arc* and *Npas4*) in RSC (C, n = 8) and HC (D, n = 6). Mean ± s.e.m. are shown. Significant differences from vehicle control are indicated by an asterisk (*).

Engagement of NMDA-R is a prerequisite for long-lasting plasticity in the HC and encoding of memory^24^, and activity-dependent gene-expression (for review, see ref.^25^). To establish if IEG-induction in RSC is linked to learning, we blocked NMDA-receptors pharmacologically. A low dose of MK801 (0.1 mg/kg) given prior to cFC training impairs memory when tested 24 hrs later (t (14) = 5.07, p < 0.001), but it only causes a tendency in reduction of freezing when tested after 1 hr (t (14) = 1.86, p = 0.08; Fig. 3b). This dose of MK801 has only subtle effects on baseline locomotor activity prior to conditioning, and it does not alter the response to the foot shock (Fig. 3a). The MK801-induced long-term memory impairment coincides with a significant impairment of IEG induction in RSC 30 mins after training (two-way interactions: *Fos* F (1, 28) = 10.98, p < 0.01; *Arc* F (1, 28) = 6.06, p < 0.05; *Npas4* F (1, 28) = 25.28, p < 0.0001; contrast: p < 0.01 for all three genes in MK801 vs. vehicle treated trained mice; Fig. 3c), as well as in HC (interactions: F (1, 20) = 6.01, p < 0.05;; *Arc* F (1, 20) = 3.86, p = 0.06; *Npas4* F (1, 20) = 8.11, p < 0.01; contrast: p < 0.01 for all three genes; Fig. 3d). Drug-injection alone (vehicle or MK801) in the absence of training caused an approximately 2-fold increase in the expression of *Fos* and *Arc*, but not *Npas4*, in both the RSC and HC. In combination with our previous results, this suggests that RSC and HC mount indistinguishable NMDA-R-dependent transcriptional responses after training, which are linked to learning.

### Memory is modulated by cAMP-signaling in RSC and HC

**Figure 4:**
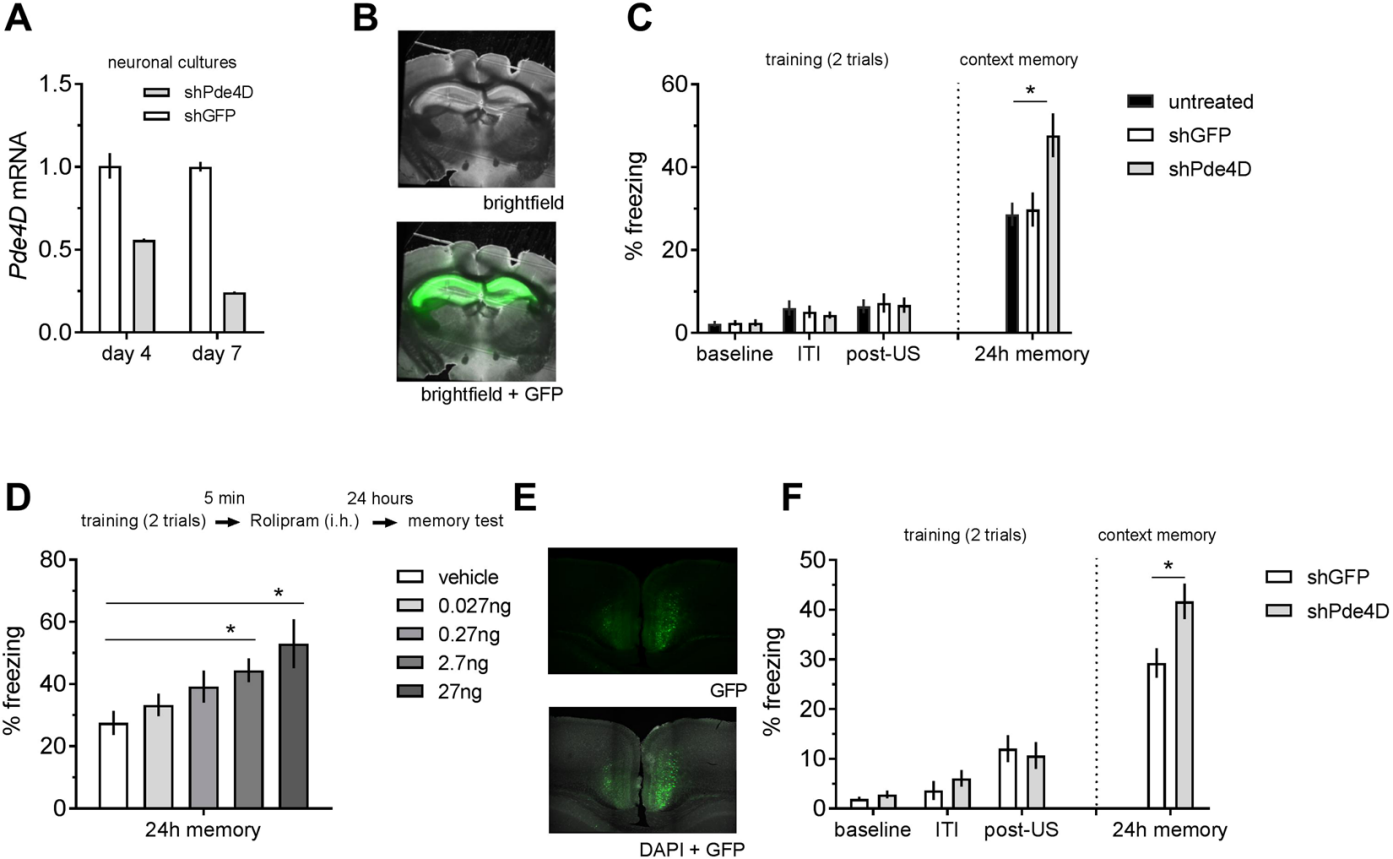
*Pde4d* knockdown (KD) in HC and RSC enhances contextual memory. mRNA levels of *Pde4d* in hippocampal cultures treated with AAV5 expressing shPde4d or shGFP control. shRNA efficiently knocks down *Pde4d* mRNA levels in cultured neurons. (**B**) Stereotactic injection of AAV5 coexpressing the shRNAs and eGFP marker results in GFP fluorescence restricted to HC. (**C**) Freezing in the memory test but not during training (shown here as baseline - before first shock, inter trial interval (ITI) - between the two shocks, and post-US – after second shock) is significantly increased after *Pde4d* KD (n = 19) in HC as compared to control virus (n = 20) and uninjected mice (n = 32). (**D**) Posttraining intrahippocampal (i.h.) injection of the PDE4 inhibitor rolipram enhances contextual memory in a dose dependent manner (n = 25 vehicle, n = 8–16 drug, respectively). (**E**) Stereotactic injection of AAV5 carrying the shRNAs and expressing eGFP results in restricted fluorescence in rostral RSC. (**F**) Freezing in the memory test but not during training is significantly increased after *Pde4d* KD (n = 23) in RSC as compared to control virus (n = 24). Significant differences from shGFP (panel C/E) or vehicle (panel D) control are indicated by an asterisk (*).

These findings suggested learning-induced plasticity in the RSC resembling that in the HC. To test this, we examined the impact of modulating mechanisms of plasticity described in HC in the RSC. We targeted the cAMP pathway, which is involved in transducing NMDA-R and neuromodulatory inputs into a transcriptional response and has been extensively characterized in hippocampus^26–29^. In this pathway, cAMP drives CREB-dependent gene transcription, a key contributor to the formation of long-term plasticity and memory^30^. cAMP can be increased specifically by blocking phosphodiesterase 4 (PDE4), which results in memory enhancement^31,32^. Since *Pde4d* is highly expressed in RSC as well as HC^33^ we considered it an ideal target to establish the contribution of cAMP signaling in each of these regions to cFC memory. For restricted PDE4 inhibition, we designed a specific shRNA against *Pde4D* mRNA and packaged it in AAV5. In cultured hippocampal neurons, this shRNA reduces *Pde4D* mRNA levels by 45% and 75% after 4 and 7 d respectively (Fig. 4a), but it had no effect on the expression of *Pde4A* and *Pde4B* (data not shown). When stereotactically injected into mouse HC, the fluorescence produced by the GFP marker coexpressed with the shRNA is found in all hippocampal subfields but not outside of the hippocampus (Fig. 4b). Mice injected in this way display normal freezing during acquisition of cFC (Fig. 4c). However, ANOVA revealed a significant main effect of treatment on freezing in the 24 hr test (F (2, 68) = 6.98, p < 0.01). Pde4D*-*shRNA injected mice froze significantly more than those injected with shGFP control virus (p < 0.05, contrast analysis) and unaltered mice (p < 0.01), while no difference was found amongst controls (Fig. 4c). Thus, *Pde4D* KD in hippocampus enhances long-term cFC memory, in addition to its previously described effect in other tests of memory^18^. To more selectively test the role of Pde4 in consolidation, we injected the selective PDE4 inhibitor rolipram into HC after cFC training and thereby circumvented acquisition. Rolipram dose-dependently enhances contextual memory (F (4, 65) = 4.358, p < 0.01; significant effect of 2.7 ng and 27 ng vs. vehicle, Dunnett’s multiple comparison test; Fig. 4d). Thus, PDE4 in the HC constrains memory consolidation which is enhanced after pharmacological inhibition or knockdown of *Pde4D*.

**Figure 5:**
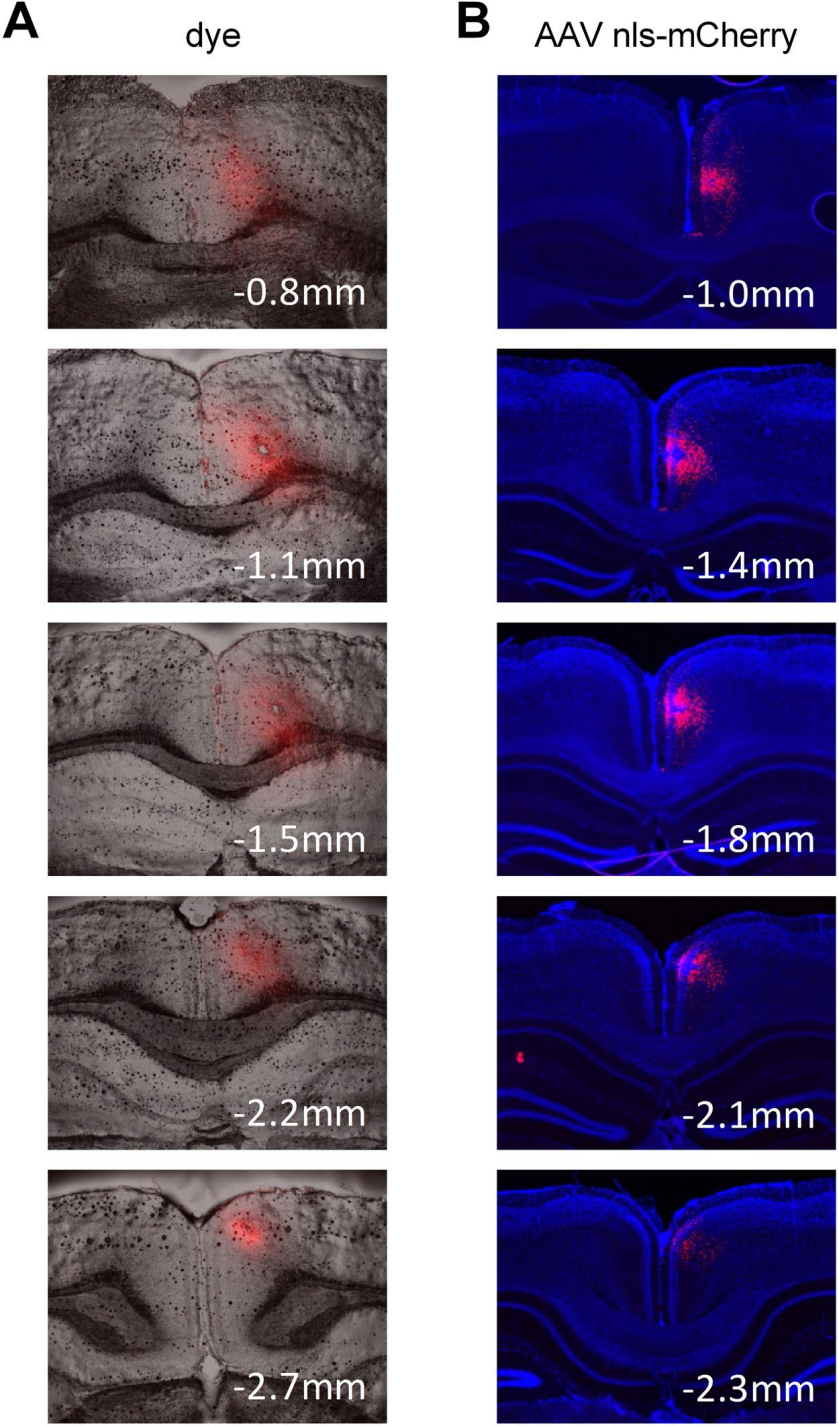
AAV virus transduction in RSC is achieved along the rostrocaudal axis. Schematic of stereotactic injection into RSC. Using this injection method, the rostrol RSC is targeted both with dye (**A**) and AAV virus expressing a nuclear localized mCherry marker (**B**, shown here for unilateral injection).

If cAMP-dependent plasticity mechanisms are similarly engaged in RSC, then targeting *Pde4D* in RSC via AAV5 mediated shRNA KD should recapitulate the memory enhancement observed in HC. To this end, we developed an injection method that would cover a large portion of the rostral RSC without leaking into adjacent cortical regions or the HC (Fig. 5). Virus injected with this method selectively infects cells in the RSC (Fig. 4e and Fig. 5b). As in HC injected mice, RSC injected mice display normal freezing during acquisition but an increase in freezing in the 24 hr test (t (45) = 2.32, p < 0.05; Fig. 4f). Because *Pde4D* KD strengthens contextual memory irrespective of whether HC or RSC is targeted, cAMP-dependent mechanisms of plasticity are critical in both areas.

### *Pde4D* knockdown induces spine maturation in RSC independent of training

**Figure 6:**
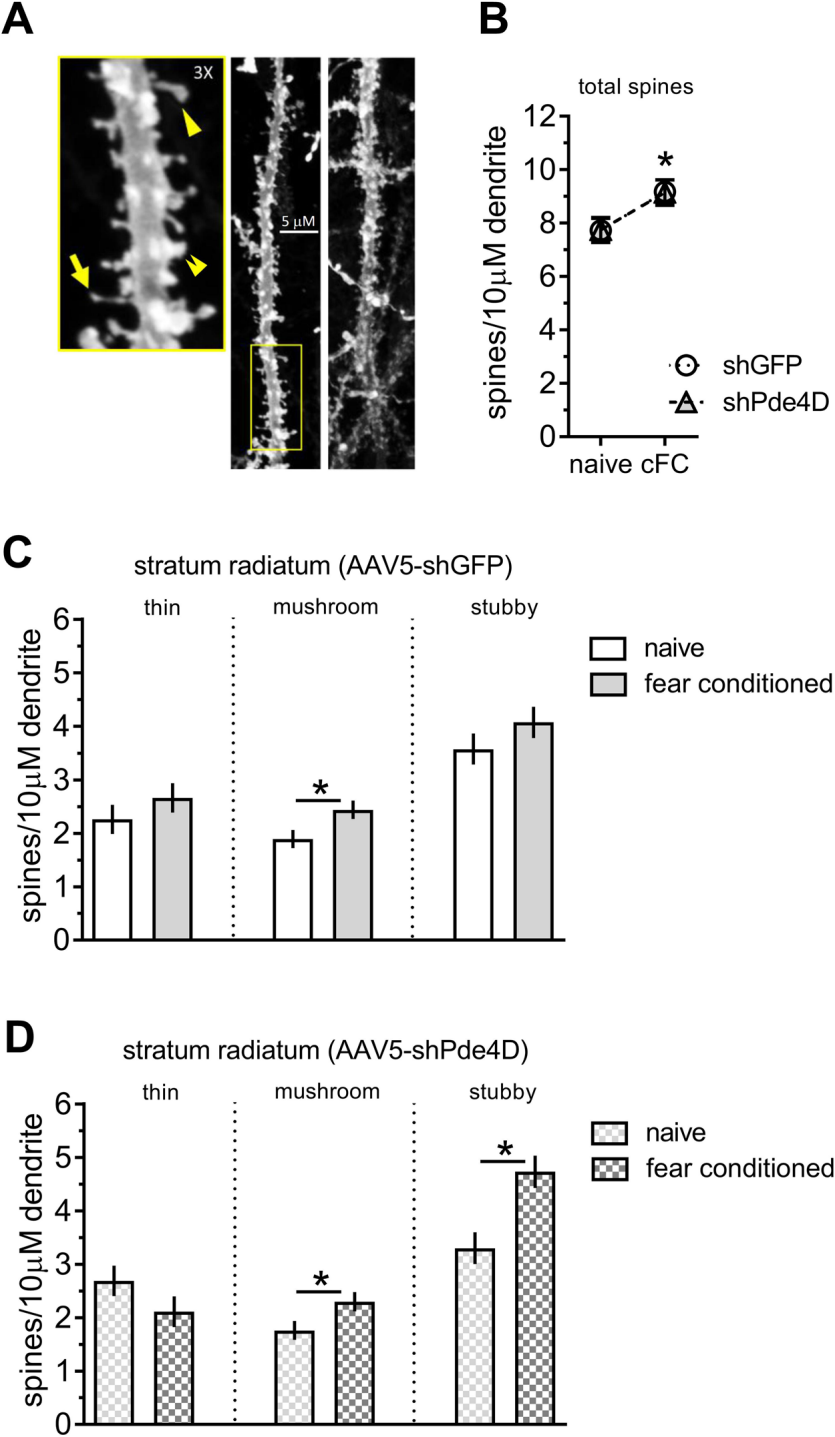
Effect of *Pde4d* KD and fear conditioning on spines in hippocampal CA1. (**A**) Representative laser-scanning confocal micrographs of dorsal HC stratum radiatum used for spine analysis. Dendritic segments of CA1 pyramidal neuron secondary apical branches were sampled 0–50 μm from branchpoint. Examples of thin-necked spines (yellow arrow), mushroom (arrowhead), and stubby (double arrowhead) spines in the 3× magnification inset represent the different spine classes. (**B**) Total spine number is increased by training independent of *Pde4d* KD. (**C**) The training induced increase mainly involves mushroom spines in the shGFP control virus injected HC. (**D**) The training induced increase involves mushroom and stubby spines after *Pde4d* KD. n = 47–52 cells from 7–8 mice per group. Significant differences from untrained mice (naive) are indicated by an asterisk (*).

To investigate the conservation of known mechanisms underlying memory consolidation further we examined the addition of new spines^34^. Spine changes within hours after training have been shown to be dynamic and transitory (for review, see ref.^35^). However, spine changes at later time points are thought to be more permanent and relevant to long-term memory. We therefore evaluated spines and the effect of *Pde4D* KD 24 hrs after training, where we previously observed memory enhancement. To understand the significance of such changes in our paradigm we first examined training-related spine changes in HC (Fig. 6) with and without *Pde4D* KD. A two-way ANOVA revealed a training-related increase in the total number of spines in stratum radiatum, independent of *Pde4D* KD (effect of trial: F (1, 194) = 9.38, p < 0.01; effect of gene-target and interaction: not significant; Fig. 6b). This increase is exclusively found in mushroom and stubby spines, but not in immature thin spines. In the HC of control virus injected mice, only mushroom spines are significantly more abundant with training (mushroom spines: p < 0.05, stubby spines: p = 0.23; planned comparison of naïve vs. trained mice; Fig. 6c). After *Pde4D* KD however, mushroom spines and mature stubby spines are significantly more abundant (mushroom spines: p < 0.05, stubby spines: p < 0.001; Fig. 6d).

**Figure 7:**
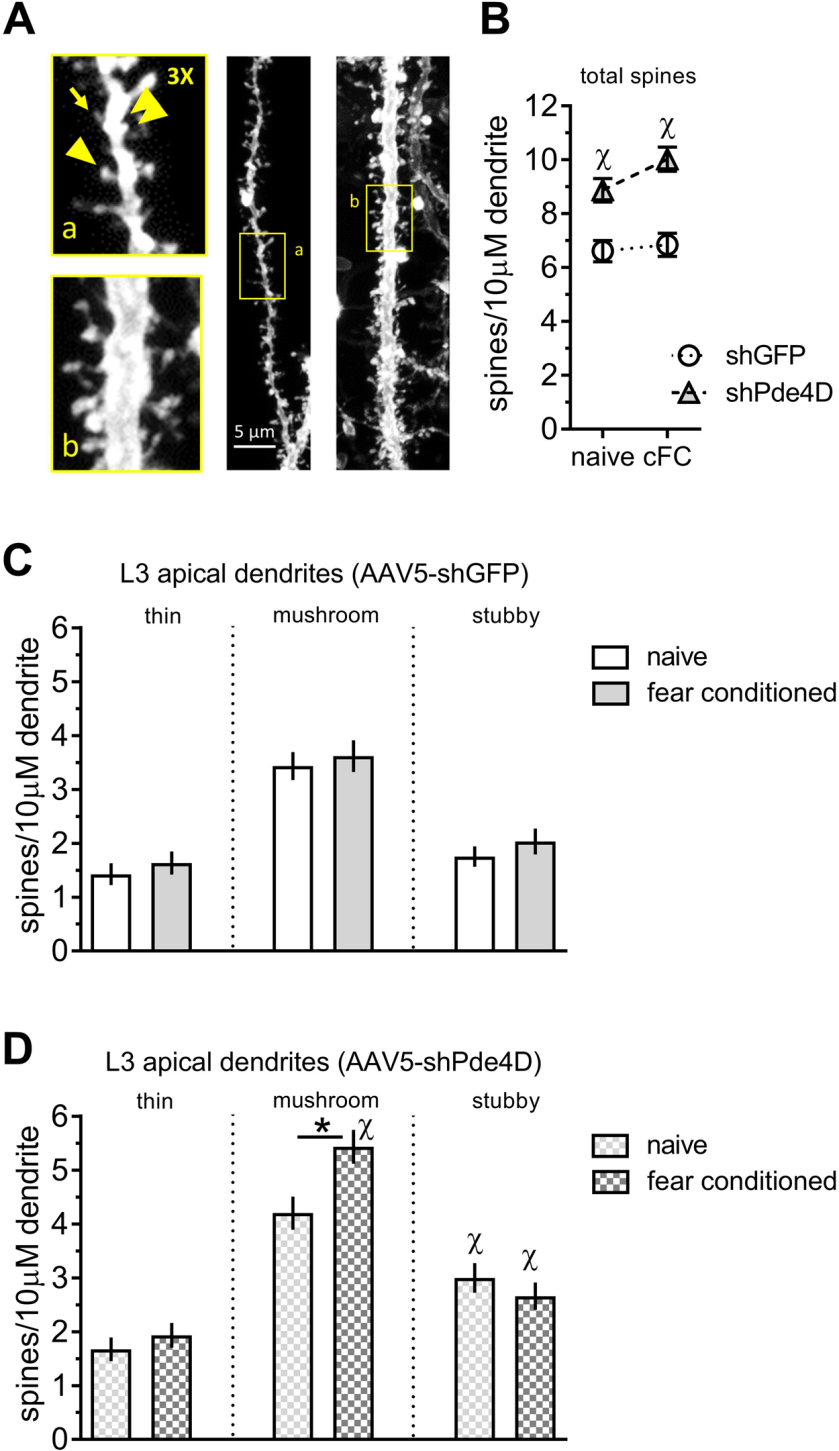
Effect of Pde4d KD and fear conditioning on spines in retrosplenial layer 2/3 neurons. (**A**) Representative laser scanning confocal micrographs of dendritic segments and dendritic spines in RSC L2/3 pyramidal neurons. Examples of thin-necked spines (yellow arrow), mushroom (arrowhead), and stubby (double arrowhead) spines in the 3× magnification insert represent the different spine classes. (**B**) Total spine number is increased by *Pde4d* KD independent of training. (**C**) Fear conditioning did not increase spine density in shGFP control virus injected mice. (**D**) Increased density of mushroom spines is observed after *Pde4d KD* and training. Stubby spines, in contrast, occur at a higher density in shPde4d virus injected mice irrespective of training when compared shGFP controls shown in panel C. n = 44–56 samples from 8 mice per group. Significant differences from untrained mice (naive) mice are indicated by an asterisk (*), and changes from shGFP control by the greek letter Chi (χ).

**Figure 8:**
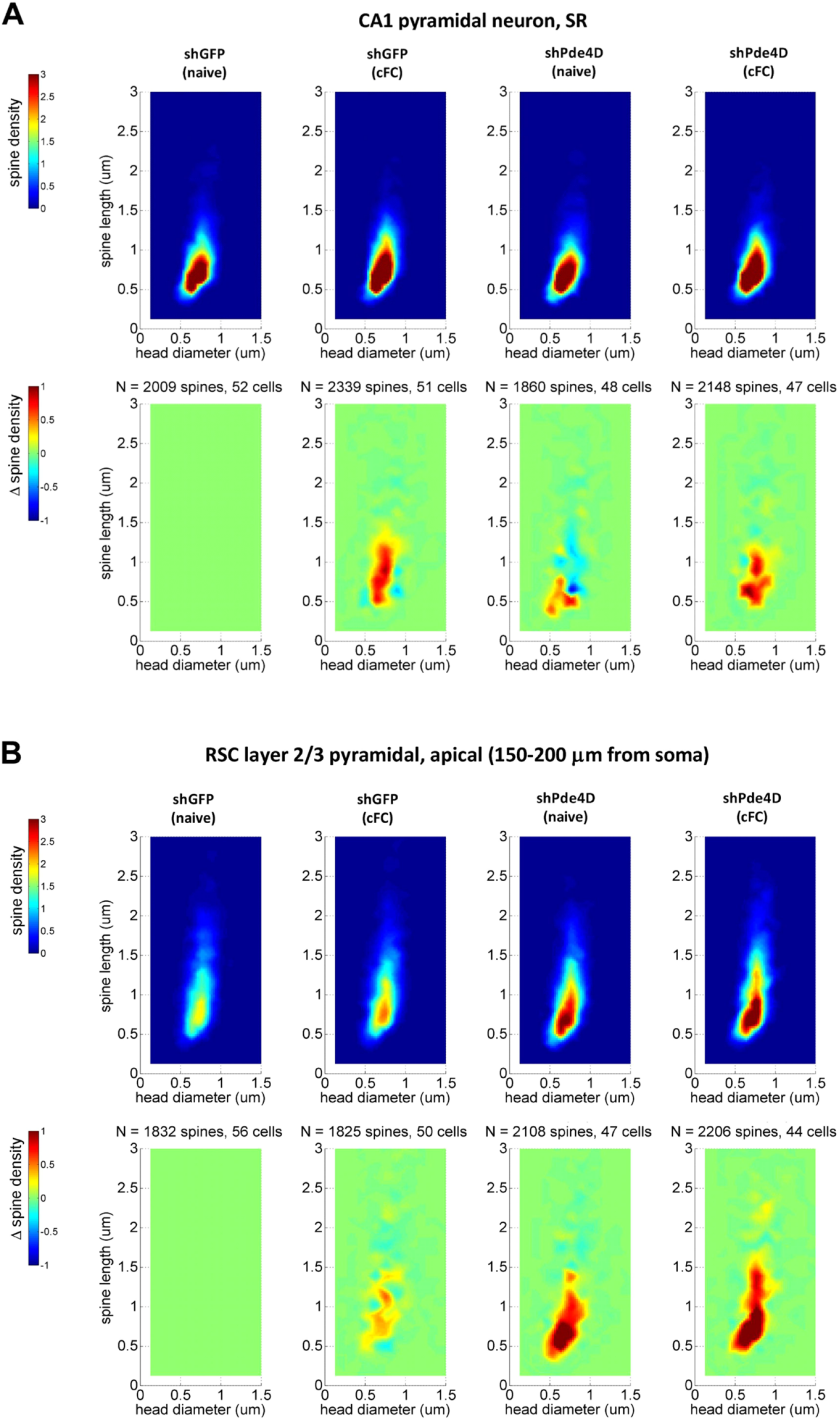
Pde4d KD and context learning modify multidimensional spine density in HC and RSC. Multidimensional spine density histograms of HC stratum radiatum (**A**) and RSC layer 2/3 apical (**B**) dendrites were derived by plotting spine densities as a function of spine length x head diameter for each sample (50 µm dendritic length) across a 2D grid at intervals of 0.125 µm for each measure followed by interpolation. Group values (spine density) represent the grid mean values (per 10 µm dendrite) for all cells tested per group (top of panel A/B). Difference plots (Δ spine density, bottom of panel A/B) were derived by comparing group mean values for each group versus shGFP (naïve). Note changes in RSC observed in trained shGFP controls, which are not captured with the classical categorization of spines into thin, mushroom, and stubby spines shown in Fig. 7.

We next investigated the differential effects of training and *Pde4D* KD on spine density in distal apical dendrites (150–200 μm from soma) of layer 2/3 neurons in the RSC (Fig. 7). The layer 2/3 neurons are positioned in the superficial layers of cortex and project highly branched apical dendrites towards the cortical surface which receive corticocortical (associational) inputs, and have been found to undergo experience-dependent modification of spines^36^. In contrast to HC, training does not significantly change overall spine number in RSC apical dendrites. Instead, *Pde4D* KD significantly increases the total number of spines independent of training (Effect of gene-target: F (1, 191) = 40.16, p < 0.0001; effect of training and interaction: not significant; Fig. 7b). This increase selectively involves mature spines, similar to the training-induced spine increase in HC (Fig. 7c/d) and is seen in both naïve and trained mice (homecage control: p < 0.0001, trained: p < 0.05; contrast analysis of shGFP vs. shPde4D treated mice). ANOVA of stubby spine density revealed a significant main effect of *Pde4D* KD (F (1, 191) = 10.66, p < 0.01; effect of training and interaction: not significant). In contrast, mushroom spine density is selectively increased after *Pde4D* KD in trained mice only. A two-way ANOVA of mushroom spine density revealed a significant main effect of training (F (1, 191) = 4.61, p < 0.05) and gene-target (F (1, 191) = 22.77, p < 0.0001), along with an interaction thereof (F (1, 191) = 4.47, p < 0.05). Furthermore, contrast analysis revealed significantly more mushroom spines in trained shPde4D mice when compared to the untrained (p < 0.01; Fig. 7c), but not in shGFP controls (p = 0.98). Thus, the effect of training on mushroom spines in RSC is augmented when cAMP-signaling is enhanced by *Pde4D* KD. Importantly, the spine changes described herein are independent of any classification. Using a distribution based depiction of the differences with treatment, we continue to observe a shift towards larger volumes and shorter necks with *Pde4D* KD and training in HC (Fig. 8a) and RSC (Fig. 8b).

Since gene expression after training are conserved between HC and RSC, but spine changes are not, our results suggest that the two may undergo different plasticities. To further investigate this, we performed a spine study using the five-trial cFC training on unaltered mice used for the gene expression study. Here again, we saw no effect of training on mature spines in the RSC (data not shown). We therefore conclude that gene expression changes are not necessarily predictive of long-lasting structural plasticity 24 hours after training.

Taken together, our results highlight several parallels but also differences in consolidation mechanisms between HC and RSC. They suggest that memory enhancement through Pde4 inhibition involves both HC and RSC. They also corroborate a previous report^5^ demonstrating the feasibility of enhancing memory by molecular manipulations in RSC, and describe structural plasticity that coincides with this enhancement. Importantly, they dissociate gene expression from structural plasticity and show differential induction of the latter between HC and RSC.

## Discussion

Here, we have shown that (i) contextual learning induces a strikingly similar pattern of gene-expression in HC and RSC, which is NMDA receptor dependent; (ii) augmentation of cAMP signaling by *Pde4D* KD enhances memory whether targeted to HC or RSC; (iii) spine-changes coincide with memory enhancement in HC and RSC; but (iv) training-induced spine changes only occur in HC. The data presented here provide evidence for engagement of RSC during memory formation, but question it as a site of learning induced plasticity.

Our findings are important for three reasons:

(A) They examine multiple processes linked to memory consolidation in two brain regions, and compare the effect of manipulations of these processes in a well-studied task. These are established processes in HC that we each corroborate in our model. Our results in RSC can therefore be interpreted in the context of vast literature around hippocampal plasticity and function.

(B) By testing processes in HC and RSC in the same model we can directly compare the findings. For instance, the gene expression changes described in Fig. 1 occur in the same mice with the same temporal profile. This allows us to compare mRNA-induction between the two regions directly and to conclude that learning induces highly correlated expression changes in RSC and in the HC. In contrast, spine changes induced by training are not mirrored in the two regions. Because we evaluate all processes in the same model we can additionally draw conclusions about their interdependence. Thus, our data suggest that training-induced, NMDA-R dependent gene expression and lasting structural plasticity co-occur in HC but not in RSC. In fact, Leuner *et al*. have described that structural plasticity depends on NMDA-R dependent gene expression in HC^37^. Our RSC data may propose that the reverse is not true, NMDA-R dependent gene expression does not predict lasting spine changes. However, we measured gene expression and spine changes in restricted time windows. It is conceivable that the timing of these processes is different in the two brain regions. This has been reported for the protein synthesis requirement of memory. The time-course of amnesia after protein synthesis inhibition in RSC^13^ is delayed as compared to the HC^38,39^. This may suggest that spine changes are delayed after fear conditioning in RSC, similar to anterior cingulate cortex^40^. However, this is contrasted by the IEG induction described herein and the findings could only be explained if the early transcriptional response in RSC and the late protein synthesis requirement are not temporally successive. Additional waves of transcription late during consolidation or local protein synthesis may explain the differential in the timecourse. Alternatively, the dissociation of transcriptional and structural changes could be explained by transient spine changes in RSC. These could rewire the neuronal circuitry in RSC without net changes in spines detected 24 hrs later. Finally, changes in synaptic weight may occur independent of changes in spine-density. For instance, mushroom spines have been described to be the target of newly synthesized AMPA receptors (AMPA-R) in CA1 within 24 hours of contextual learning^41^. Such trafficking of AMPA-R at postsynaptic sites is a key determinant of synaptic strength and plasticity^42,43^. An AMPA-R relocalization study in RSC could highlight changes in connectivity in RSC that occur independent of spine changes. However, the scope and complexity of such experiments renders this subject of a future study. Additionally, homeostatic spine modulation may help to obfuscate spine modifications after training, a phenomenon observed in at least some neuropsychiatric conditions^44,45^, although we would expect the effect to be limited based on the duration of the training period.

(C) Our data also suggest that systemic *Pde4D* manipulation may enhance memory through regulating functionally different plasticity in HC and RSC. In HC, *Pde4D* KD mediates memory enhancement and augments spine changes occurring physiologically with training. Such augmentation has previously been reported to coincide with memory enhancement. For instance stress-induced memory enhancement in cFC coincides with a higher density of “mature” CA1 spines, a category that includes stubby and mushroom spines^46^. Similarly, memory enhancement by PKCalpha activation increases mushroom-type, mature spines in CA1 after training in the Morris water maze^47^. More generally, acquisition of a strong memory can elevate the number of hippocampal dendritic spines in diverse memory tasks including fear conditioning^37,40^. In RSC however, there are no spine changes observed 24 hours after learning, even when using a strong training paradigm. Instead, *Pde4D* KD appears to induce a state of metaplasticity that supports enhanced memory by adding stubby spines (also see ref.^48^), and it enables the addition of mushroom spines within 24 hrs of training. Such state may also be achieved by overexpression of CREB in RSC. CREB overexpression in a subpopulation of RSC neurons, like *Pde4D* KD, enhances spatial memory. Silencing these neurons before memory test occludes memory enhancement but not the recall and reverts memory performance to control levels^5^. Different states of metaplasticity may also be at the heart of other enhancement methods, such as environmental enrichment. Moser *et al*. showed that chronic exposure to a spatially complex environment increased spine density in CA1 and accelerated acquisition in the Morris water maze^49^.

How could structural metaplasticity in RSC promote memory? The increases in spine number in this study involve mature spines and this is expected to have a large impact on connectivity. Mature spines - in particular stubby spines - boast larger volumes, shorter necks and more synaptic machinery than other spine types; features that ensure greater responses to synaptic activity with larger dendritic depolarization^50^; (for review, see ref.^51^). They are also more likely to be stable spines (for review, see ref.^52^) and as such are able to provide the cellular and network framework for long-term memory^53^. It is unclear whether the spine changes occur at specific projections innervating RSC neurons. However, previous work has established that RSC activity requires HC input^4,54,55^. HC inactivation impairs natural memory recall but artificially recapitulating the RSC activity that was active during training elicits the conditioned response^17^. This suggests that the HC may instruct activity in RSC to facilitate memory recall. In turn, hippocampal “place fields” are affected by temporary inactivation of retrosplenial cortex^56^ indicating some opposite control as well. Metaplastic spine changes may improve communication between RSC and HC and generate more synchronous activity. In humans, decreased resting state activity in the RSC of aMCI (amnestic mild cognitive impairment) patients has been described^57^. Moreover, activation differs between MCI and age matched controls during memory recall and this has been hypothesized to reflect local functional reorganization^58^. We suggest that augmenting cAMP–signaling improves hippocampal plasticity in response to learning and causes metaplasticity in cortical circuits, including the RSC. Increased cAMP-signaling and behavioral training may work in concert to add synaptic connectivity across brain circuits, thereby providing a basis for the therapeutic use of PDE4 inhibitors in the treatment of memory disorders.

### Significance statement

The retrosplenial cortex (RSC) has been of considerable interest due to the severity of retrosplenial amnesia in humans. The nature of its involvement in memory is however controversial and the mechanisms governing memory in the RSC have remained largely unexplored. We describe experiments that systematically interrogate core mechanism of memory consolidation such as activity-dependent gene expression, reliance on cAMP signaling, and spine maturation in the RSC. Our findings reveal that multiple mechanisms of memory that have been well characterized in HC are conserved in the RSC. We show that memory enhancement with PDE4 inhibition is likely mediated through both HC and RSC, but that RSC may not undergo the same physiological plasticity as HC.
